# Pulsed electromagnetic fields vs NSAID therapy in canine osteoarthritis: A randomised comparative pilot study

**DOI:** 10.17221/54/2025-VETMED

**Published:** 2026-03-12

**Authors:** Sara Sassaroli, Fabrizio Dini, Valentina Sisti, Valentina Riccio, Silvia Meggiolaro, Linda Bellodi, Angela Palumbo Piccionello

**Affiliations:** ^1^School of Biosciences and Veterinary Medicine, University of Camerino, Matelica, Italy; ^2^Veterinary Freelance, Abano Terme, Italy

**Keywords:** canine rehabilitation, degenerative joint disease, joint mobility, magnetotherapy, pain assessment

## Abstract

Non-steroidal anti-inflammatory drugs (NSAIDs) are a cornerstone in the management of canine osteoarthritis (OA), despite concerns regarding their long-term safety. Among non-pharmacological alternatives, pulsed electromagnetic field (PEMF) therapy has gained attention for its potential analgesic and anti-inflammatory effects, although veterinary-specific evidence remains limited. This randomised, controlled pilot study compared the clinical efficacy of PEMF therapy versus NSAID treatment (Mavacoxib) in 16 dogs with clinically and radiographically confirmed OA. Dogs were randomly assigned to receive either a 12-session PEMF protocol over 45 days or a standard Mavacoxib regimen. Clinical evaluations included pain scores (VAS), pain on palpation, lameness severity (NRS), gait analysis (GLS and TPI), muscle circumference, and radiographic progression. Assessments were performed at baseline (T0), 30 days (T1), and 60 days (T2). PEMF therapy showed earlier improvements in pain, lameness, and muscle mass, and a favourable trend in joint mobility. No significant differences were detected in gait or radiographic parameters between groups. These preliminary findings suggest that PEMF is non-inferior to NSAIDs and highlight its potential role in multimodal OA management. Limitations include a small sample size and a short follow-up. Further studies are needed to confirm these preliminary results in larger cohorts.

Osteoarthritis (OA) is a prevalent and painful degenerative disease that affects joint cartilage (Kulkarni et al. 2021; Graves et al. 2023). Joint tissue remodelling is not merely a consequence of mechanical wear but rather an abnormal process influenced by various predisposing factors (Anderson et al. 2020; Graves et al. 2023). These factors include age (being more common in older individuals), sex, previous injuries, obesity, level of physical activity, environmental influences, genetic predispositions (such as elbow or hip dysplasia), mechanical factors like misalignment (leading to abnormal and unevenly distributed loads), and joint instability (Guilak 2011).

Osteoarthritis is associated with an imbalance in joint homeostasis that favours catabolic processes. This disruption in joint homeostasis may result from metabolic, inflammatory, or biomechanical dysfunction. Although OA has a multifactorial aetiology, its pathological manifestations share common features, including degradation of articular cartilage, sclerosis of the subchondral bone, formation of osteophytes and intra-articular mineralisation, synovial and ligamentous inflammation, and hypertrophy of the synovial capsule (Damyanovich et al. 1999; Braun and Gold 2012). The pathology clinically manifests with intermittent joint lameness and stiffness (often worsening after inactivity, improving with mild exercise and deteriorating after strenuous activity), pain, reluctance to move, easy fatigability, joint swelling, loss of function accompanied by crepitus, reduced range of motion (ROM), and muscle atrophy, all of which severely impact the patient’s quality of life. Assessment tools can quantify these aspects, facilitating classification of the pathological process and monitoring clinical progress, particularly during treatment (Hielm-Bjorkman et al. 2003; Lascelles et al. 2006).

There is no definitive cure for osteoarthritis; as it is a progressive and degenerative condition, the main treatments aim to relieve pain and inflammation, improve mobility, and slow the progression of the disease, typically through a conservative, multimodal approach (Marcellin-Little et al. 2025).

In severe cases, surgical therapy may be necessary, including prosthetic joint replacement (e.g., hip joint), arthrodesis, and curettage.

Among conservative therapies, the use of non-steroidal anti-inflammatory drugs (Papich and Messenger 2015; White and Morrow 2020; Tellegen et al. 2023) is currently the most widely used. New formulations have been developed in recent years to manage chronic OA pain, including cannabinoids (Brioschi et al. 2020) and monoclonal antibodies (Enomoto et al. 2019). Other pharmacological classes that can be useful in the treatment of OA are analgesics such as amantadine, gabapentin, tramadol, and buprenorphine, and infiltrative therapies with corticosteroids, hyaluronic acid, stanozolol, and, in the field of regenerative medicine, with platelet-rich plasma (PRP) (Raeissadat et al. 2020; Alves et al. 2021) and mesenchymal stem cells (MSCs) (Sanghani-Kerai et al. 2021).

Conservative non-pharmacological therapies play a key role in OA management and include weight control (Pye et al. 2024), nutritional support (Comblain et al. 2017), and rehabilitative physiotherapy. The physiotherapy includes both manual techniques (Millis and Levine 2014), such as active exercises, passive mobility techniques (including kinesiotherapy and exercise therapy) and massage therapy, and instrumental techniques, such as hydrotherapy, laser therapy, transfer of energy capacitive and resistive therapy (TECAR), ultrasound, electrostimulation, shock waves, and magnetotherapy (Pinna et al. 2013; Sullivan et al. 2013; Gaynor et al. 2018).

Magnetotherapy, also known as pulsed electromagnetic field therapy (PEMF), is a technique that utilises electromagnetic fields to influence tissue repair processes, regulate cellular electrochemical balance, and reduce inflammation and pain (Gomez-Ochoa et al. 2011; Varani et al. 2017). PEMF therapy is indicated for the treatment of osteoarthritis, pain, fractures, wound healing, inflammation, and post-operative oedema. Unlike NSAIDs, PEMF has been proven to be a safe therapy with no side effects (Monteiro-Steagall et al. 2013).

While NSAIDs remain a cornerstone in managing canine osteoarthritis, their long-term use is often limited by their gastrointestinal, renal, and hepatic side effects. Pulsed electromagnetic field (PEMF) therapy, with its non-invasive nature and minimal reported adverse effects, has emerged as a promising alternative that may overcome these limitations.

## Study objective

This study aimed to directly compare the efficacy and safety of PEMF therapy with a standard NSAID regimen in a controlled clinical setting.

## MATERIAL AND METHODS

### Ethical statement

The study was conducted in accordance with Italian and European rules on animal welfare. All dog owners were informed about the study’s purpose and provided written consent before enrolment and data collection.

### Study design

The present study was a single-centre, prospective, interventional, randomised clinical trial. The study was offered to all eligible patients visiting the facility over a 12-month period. Patients were randomly assigned to the NSAID or PEMF groups using a computer-generated random number table, ensuring equal allocation and reducing selection bias.

No blinding of owners or outcome assessors was implemented, which may have introduced subjectivity into some of the evaluated parameters.

### Study population

Canine patients diagnosed with osteoarthritis in any joint were recruited based on specific inclusion and exclusion criteria.

The inclusion criteria were an ASA score of 2 or lower, age between 1 and 10 years, and body weight between 5 and 85 kg. There were no restrictions based on sex or breed. The presence of lameness (due to joint disease), classified using the NRS scale, and a radiographic OA grade between 1 and 4, assessed using the modified Kellgren–Lawrence scale, were also required for inclusion.

Patients were excluded if they had poor general health, were pregnant or lactating, or had unconsolidated joint fractures, joint instability, or intra-articular loose bodies. Additionally, subjects that had received anti-inflammatory drugs, analgesics, or corticosteroids within 15 days prior to enrolment were not eligible. Dogs with osteoarthritis lesions affecting two or more joints of the same limb were also excluded.

### Treatment procedures

Patients in the PEMF group underwent 12 therapy sessions over 45 days, with two sessions per week, each lasting 50 minutes. Treatments were administered using the PMT QS portable magnetotherapy device (ASA S.r.l, Arcugnano, Italy) with Flexa applicators. The frequency range was set to 0.5–100 Hz, and the intensity to 1.5–4 mT. The initial sessions focused on vasodilation and muscle relaxation, while later sessions aimed to achieve anti-inflammatory and biostimulatory effects. The device settings used during the treatment cycle are reported in Table 1.

**Table 1:** Protocols used during the different magnetotherapy sessions

Session	Intensity	Frequency	Magnetic field	Duration
	(%)	(Hz)	(mT)	(min)
1^st^–3^rd^				
Phase 1	40	33	1.5	25
Phase 2	70	33	2.2	25
				
4^th^–8^th^				
Phase 1	100	10	4.0	25
Phase 2	60	100	1.3	25
9^th^–12^th^	95	50	3.2	45

Hz = hertz; min = minutes; mT = millitesla; sec = seconds

Patients in the NSAID group received Trocoxil^®^ (Mavacoxib), a COX-2 selective inhibitor used for OA pain management. The dosage was 2 mg/kg at T0, with repeat doses administered at 15 and 45 days post-enrolment.

### Clinical outcomes

The effects of the two treatment approaches were assessed by measuring pain and pain on palpation, the degree of lameness, muscle atrophy, gait analysis, joint range of motion (ROM), and OA severity. Assessments were performed at baseline (T0), 30 days (T1), and 60 days (T2) post-enrolment.

Pain was evaluated using a visual analogue scale (VAS), with 0 indicating no pain and 10 the highest level of pain. Behavioural signs such as limb withdrawal, discomfort while sitting and standing, abnormal posture, vocalisation, panting, and aggression were considered when assessing scores.

Pain on palpation was assessed using a scale ranging from 1 to 4, where 1 indicated no pain and 4 represented the highest level of pain, characterised by immediate retraction of the limb by the patient with any attempt at manipulation.

Lameness was assessed by observing patients during walking and trotting, considering weight distribution, pain indicators, and gate abnormalities. The severity was scored using a numeric rating scale (NRS) ranging from 0 (no lameness) to 4 (severe lameness), as reported in Table 2.

**Table 2:** NRS scale for evaluating the degree of lameness

Walk/trot	Clinical signs
Grade 0	no evidence of lameness when walking or trotting
Grade 1	no evidence of lameness when walking; slight lameness when trotting
Grade 2	slight lameness when walking; obvious lameness when trotting
Grade 3	obvious lameness when walking; load subtraction at a trot
Grade 4	lameness with lack of support both walking and at a trot

NRS = numeric rating scale

Muscle atrophy due to OA was assessed by measuring limb circumference with a flexible tape measure while the patient was standing. Measurements were taken at the mid-arm (midpoint between the greater tubercle and the lateral epicondyle of the humerus) for the thoracic limb and at the mid-thigh (midpoint between the greater trochanter and the lateral epicondyle of the femur) for the pelvic limb. To improve reliability, the average of three measurements was calculated, and the percentage improvement between time points (T0–T1, T1–T2, and T0–T2) was used for intergroup statistical analysis.

Gait analysis was conducted using the GAIT4 Dog^®^ walkway system (CIR Systems Inc., Sparta, NJ, USA). This system recorded first and last paw contact, stride time and velocity, stance time, stride length, width, and reach, as well as peak pressure distribution among limbs. Two key parameters were analysed: the Gait Lameness Score (GLS) and the Total Pressure Index (TPI). GLS is a numerical scale used to assess the degree of lameness by analysing weight-bearing distribution across all four limbs. The scoring system is based on a 100-point scale, where lower GLS scores indicate more pronounced lameness and an asymmetrical gait pattern. A GLS score approaching 100 suggests a more balanced weight distribution and a near-normal gait. This parameter is particularly useful in detecting even subtle asymmetries that may not be evident through visual observation alone.

**Figure 1:**
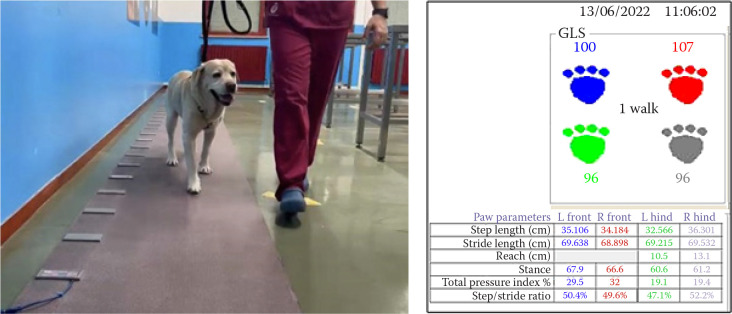
The figure presents an example of data acquisition during gait analysis. On the right, the software displays GLS and TPI, on the left, the patient is led along a walkway during data acquisition GLS = gait lameness score; TPI = total pressure index

TPI measures the proportion of total ground reaction force exerted by each limb. It is calculated by totalling the peak pressure values recorded from all activated sensors during footplate contact and then determining the percentage contribution of each limb. In a healthy dog, the expected TPI distribution is approximately 60% for thoracic limbs (30% for each forelimb) and 40% for pelvic limbs (20% for each limb). A deviation from these expected values indicates a compensatory weight shift due to pain or dysfunction in a particular limb. A decrease in TPI for the affected limb suggests reduced weight-bearing, while an increase in the contralateral limb’s TPI indicates compensatory overloading. Assessments for GLS and TPI were conducted at T0 and T2 to evaluate the changes in gait and load distribution resulting from treatment. An example of data acquisition during a gate analysis is reported in Figure 1.

Joint range of motion (ROM) was evaluated using an orthopaedic goniometer while the patients were sedated. Maximum flexion and extension values were recorded for each joint, and the mean of three repeated measurements was used. For each subject, the joint excursion angles were calculated as the difference between the measured extension and flexion angles. Due to the heterogeneity of the joints involved in the study population, absolute ROM values were normalised by comparison with joint-specific physiological reference values (Jaegger et al. 2002), as reported in Table 3. The resulting percentage deviation from the expected excursion range was computed at each time point, allowing consistent intra- and inter-group comparisons regardless of the specific joint assessed. These normalised values were then used for subsequent statistical analyses.

**Table 3:** Reference values of flexion, extension, and joint excursion for different types of articulations in dogs

Articulation	Flexion	Extension	Excursion
Shoulder	57°	165°	108°
Elbow	36°	165°	129°
Carpus	32°	196°	164°
Hip	50°	162°	112°
Tarsus	39°	164°	125°

Radiographic assessment of the affected joint was performed under sedation in ventro-dorsal, cranio-caudal, and medio-lateral projections, depending on the joint involved. OA severity was graded using the modified Kellgren–Lawrence scale, which ranges from 0 to 4. The presence of osteophytes, subchondral bone sclerosis, joint space narrowing, and capsular ectasia was considered. Radiographic evaluations for OA severity were conducted at T0 and T2 to assess baseline status and disease progression.

### Statistical analysis

Statistical analyses were performed using Python libraries. Data were expressed as mean ± standard deviation for normally distributed variables, as median (interquartile range) for non-parametric data, and as frequencies (%) for categorical variables. The Shapiro–Wilk test was used to assess normality. Between-group comparisons were conducted using independent-sample *t* tests or Mann–Whitney *U* tests, as appropriate. Within-group changes over time were analysed using repeated-measures ANOVA or Friedman tests. When significant effects were found, post-hoc comparisons were adjusted using the Holm-Bonferroni correction. Effect sizes and 95% confidence intervals were calculated, with statistical significance set at α = 0.05.

## RESULTS

Sixteen client-owned dogs were enrolled between June 2022 and May 2023 at the Teaching Veterinary Hospital of the School of Veterinary Medical Sciences in Matelica (MC), Italy. The cohort included 6 females and 10 males, aged 7–10 years, weighing 10–55 kg, with OA affecting the tarsus (*n = *3), carpus (*n = *2), elbow (*n = *3), shoulder (*n = *2), and hip (*n = *6). Subject characteristics are summarised in Table 4. No statistically significant differences were found between treatment groups in terms of age, weight, sex, or OA severity distribution (*P* > 0.05).

**Table 4:** Subject characteristics at baseline visit

Characteristics	NSAID group (*n* = 8)	PEMF group (*n* = 8)	*P*-value
	mean ± SD or *n* (%)	mean ± SD or *n* (%)	
Age (years)	8.8 ± 1.6	9.11 ± 1.0	0.158 3^b^
Weight (kg)	35.1 ± 17.9	19.8 ± 9.8	0.187 3^b^
			
Sex			
Female	4 (50)	2 (25)	0.605 6^a^
Male	4 (50)	6 (75)	
			
Breed			
Mixed-breed	6 (75)	7 (87.5)	0.214 7^a^
Setter	2 (25)	0 (0)	
Labrador	0 (0)	1 (12.5)	
			
Articulation			
Thoracic limb			0.122 3^a^
Carpus	1 (12.5)	1 (12.5)	
Elbow	2 (25)	1 (12.5)	
Shoulder	2 (25)	0 (0)	
		
Pelvic limb			
Hip	0 (0)	6 (75)	
Tarsus	3 (37.5)	0 (0)	
			
Articulation type			
Thoracic	5 (62.5)	2 (25)	0.313 5^a^
Pelvic	3 (37.5)	6 (75)	
			
Osteoarthritis stage			
Grade 1	4 (50)	2 (25)	0.513 4^a^
Grade 2	0 (0)	0 (0)	
Grade 3	2 (25)	2 (25)	
Grade 4	2 (25)	4 (50)	

^a^Chi-square test; ^b^Wilcoxon Mann–Whitney *U *test (two-tailed)

NSAID = non-steroidal anti-inflammatory drugs; PEMF = pulsed electromagnetic field; SD* = *standard deviation

### Pain and pain at palpation

VAS pain scores decreased over time in both groups, but significantly only in the PEMF group (Friedman *P = *0.000 6). Wilcoxon post-hoc analysis confirmed significance between T0–T1 and T0–T2, with median values showing a consistent drop. In the NSAID group, no timepoint comparison reached significance. Both groups showed improvement on palpation at T1, but only the PEMF group maintained reduced scores through T2. These effects suggest a more durable analgesic response with PEMF. The results are presented in Table 5.

**Table 5:** Clinical outcomes evaluated during the study and analysis within and between NSAID and PEMF groups

Clinical outcomes	NSAID group	PEMF group	*P*-value (between group)	Effect size	95% CI for *d*
	(mean ± SD)	(mean ± SD)	(*P*)^b^	(Cohen’s *d*)	
Pain					
T0	6.3 ± 2.6	6.8 ± 2.4	0.631 4	–	–
T1	5.6 ± 2.2	5.3 ± 2.1	0.472 1	–	–
T2	5.0 ± 2.0	4.8 ± 2.1	1.000	+0.10	[–0.88, +1.08]
*P* ^a^	0.041 8	0.000 6	–	–	–
					
Pain at palpation					
T0	1.8 ± 0.9	2.6 ± 1.0	0.113 3	–	–
T1	1.0 ± 0.8	1.6 ± 1.0	0.243 9	–	–
T2	1.3 ± 0.9	1.6 ± 1.0	0.545 2	–0.32	[–1.30, +0.67]
*P* ^a^	0.009 4	0.000 3	–	–	
					
Muscle circumference					
T0	19.9 ± 6.2	27.4 ± 5.0	–	–	–
T1	20.3 ± 7.0	30.1 ± 5.2	–	–	–
T2	20.3 ± 7.0	31.2 ± 4.9	–	–	–
*P* ^a^	0.449 3	0.000 5	–	–	–
Δ% (T0→T1)	1.4 ± 3.9	10.4 ± 8.3	0.012 8	–	–
Δ% (T1→T2)	–0.2 ± 1.3	4.0 ± 4.3	0.023 9	–	–
Δ% (T0→T2)	1.2 ± 3.9	14.9 ± 11.3	0.006 9	–1.62	[–2.77, –0.47]
					
GLS					
T0	91.0 ± 7.9	94.9 ± 5.9	0.461 9	–	–
T2	92.3 ± 8.1	97.6 ± 14.4	0.382 2	–0.45	[–1.45, +0.54]
*P* ^a^	0.054 7	0.742 2	–	–	–
					
TPI					
T0	24.5 ± 4.4	21.3 ± 4.7	0.115	–	–
T2	24.6 ± 4.8	21.8 ± 4.4	0.371	+0.61	[–0.40,+1.61]
*P* ^a^	0.233	0.742	–	–	–
					
Articular excursion normalised (% of ref.)					
T0	−13.2 ± 13.8%	−30.0 ± 21.1%	0.103 3	–	–
T1	−12.2 ± 11.6%	−9.1 ± 19.2%	0.699 7	–	–
T2	−12.7 ± 11.7%	+0.1 ± 16.8%	0.100 0	–0.88	[–1.92, +0.15]
*P* ^a^	0.960 7	0.002 3	–	–	–

^a^Friedman test; ^b^Mann–Whitney *U* test

CI = confidence interval; GLS = gait lameness score; NSAID = non-steroidal anti-inflammatory drug; PEMF = pulsed electromagnetic field; ROM = range of motion; SD = standard deviation; TPI = total pressure index

### Lameness

**Figure 2:**
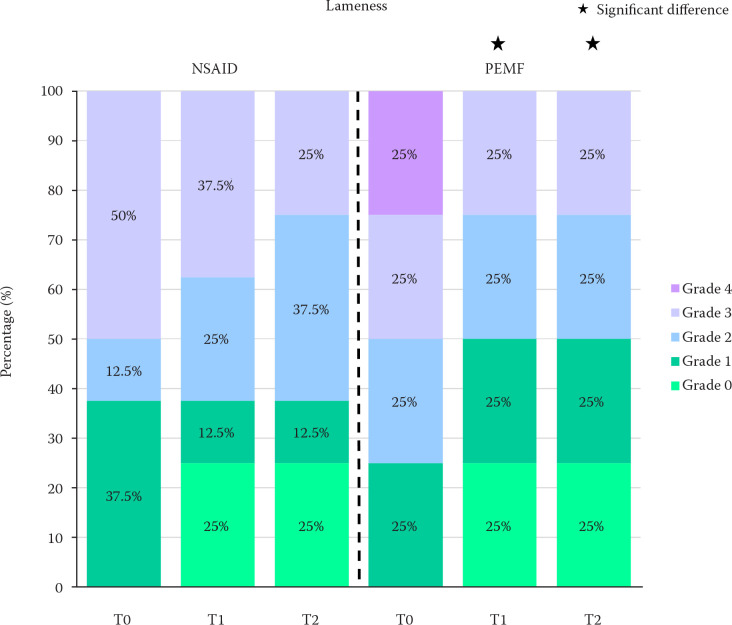
The figure illustrates the distribution of lameness severity in the two treatment groups over the course of the study NSAID = non-steroidal anti-inflammatory drug; PEMF = pulsed electromagnetic field

PEMF-treated dogs demonstrated significant reductions in NRS lameness scores from T0 to both T1 and T2 (Friedman *P = *0.000 3). No significant changes were observed in the NSAID group. Inter-group analysis showed no statistical difference, but graphical trends (Figure 2) favoured PEMF. This early and sustained improvement in locomotor function may indicate a faster onset of clinical benefit.

### Muscle circumference

Intra-group comparisons revealed no significant changes in the NSAID group, whereas the PEMF group showed marked, statistically significant increases at both T1 and T2 (Friedman, *P = *0.000 5). Between-group differences were significant across all intervals, with the PEMF group achieving a mean 14.9% increase in muscle mass from T0 to T2. This corresponded to a very large effect size (Cohen’s *d* = –1.62, 95% CI –2.77 to –0.47) favouring PEMF, suggesting improved functional use and weight-bearing of the affected limbs (Table 5).

### Gait analysis

The Gait Lameness Score (GLS) and Total Pressure Index (TPI) did not show significant changes over time in either group. In the PEMF cohort, a trend toward GLS normalisation was observed, albeit with wide inter-individual variability. At T2, between-group comparisons revealed small-to-moderate effect sizes with wide confidence intervals (GLS: Cohen’s *d* = –0.45, 95% CI –1.45 to 0.54; TPI: Cohen’s *d* = +0.61, 95% CI –0.40 to 1.61), consistent with the absence of statistically or clinically meaningful differences. Overall, these results underscore the limitations of instrumental gait analysis in detecting subtle functional changes in small pilot samples (Table 5).

### Range of motion

PEMF treatment was associated with a significant improvement in joint excursion values over time (Friedman *P = *0.002 4), with mean ROM progressively approaching reference physiological values at T2. Pairwise comparisons (Wilcoxon test with Holm–Bonferroni correction) showed near-significant gains from T0–T1 (*P = *0.053 2) and T0–T2 (*P = *0.053 8). No intra-group improvements were observed in the NSAID cohort. At the between-group level, differences did not reach statistical significance (*P = *0.10); however, the effect size was large (Cohen’s *d* = –0.88, 95% CI –1.92 to 0.15), which may indicate a clinically relevant trend favouring PEMF (Table 5).

### Radiographic severity

No OA progression was detected using Kellgren-Lawrence scoring from T0 to T2 in either group. This suggests both treatments provided effective control of disease activity over the short term.

These results, taken together, indicate that PEMF therapy is at least as effective as NSAID therapy in reducing clinical signs of OA, while also offering potential advantages in muscle preservation, ROM restoration, and early analgesic effect.

## DISCUSSION

This randomised pilot study aimed to assess the clinical efficacy of pulsed electromagnetic field (PEMF) therapy compared with a COX-2-selective NSAID (Mavacoxib) in the management of canine osteoarthritis (OA). While both treatments demonstrated beneficial effects across several outcome measures, PEMF was associated with a faster onset of clinical improvement and greater preservation of muscle mass and improved joint mobility.

The analgesic effect of PEMF was particularly evident in the early phases of treatment. Significant improvements in both VAS pain scores and lameness grades were observed as early as day 30 (T1) and remained stable at day 60 (T2). In contrast, the NSAID group failed to show sustained or statistically significant reductions in pain or lameness.

Of particular clinical interest is the increase in muscle circumference observed exclusively in the PEMF group. This outcome suggests improved functional recovery, possibly resulting from enhanced weight-bearing and decreased guarding behaviours due to pain relief. Such findings are consistent with those of Sprunks et al. (2024), who demonstrated significant improvement in quality of life and passive range of motion in dogs receiving PSWT targeting the vagus nerve. Similarly, Pinna et al. (2013) reported greater long-term efficacy of PEMF compared with firocoxib, with more durable improvements in lameness and a delayed return of symptoms. These studies, along with the present findings, support the hypothesis that PEMF may not only reduce pain but also contribute to functional recovery and tissue remodelling.

Nevertheless, in both veterinary and human medicine, studies report no significant differences between PEMF- and sham-treated groups in the treatment of osteoarthritis (Ay and Evcik 2009; Ozguclu et al. 2010; Sullivan et al. 2013). This heterogeneity underscores the need for further standardisation of trials in terms of clinical designs, PEMF types, and treatment protocols.

With regard to structural changes, this study demonstrated stability in OA progression on radiographic examination in both groups; the observed clinical improvements in the PEMF group suggest a decoupling between structural joint changes and functional disability. This phenomenon has been noted in both veterinary (Enomoto et al. 2024) and human OA literature (Hill et al. 2025) and highlights the importance of incorporating functional and quality-of-life metrics, such as lameness, pain on palpation, and muscle trophism, into therapeutic evaluations.

Gait parameters, including GLS and TPI, did not change significantly over the course of the study in either group. This may be attributed to the limited sample size, high inter-individual variability, and insensitivity of these metrics to subtle improvements in clinical status. Nonetheless, ROM analysis revealed a clear trend toward normalisation in the PEMF group, with joint excursion values progressively approaching physiological norms. Given the chronic and progressive nature of OA, such improvements in joint mobility over a relatively short observational period are encouraging and may predict better long-term outcomes.

Another key aspect to consider in comparing the treatments is safety. While Mavacoxib has a favourable pharmacokinetic profile that enables monthly administration, its potential for gastrointestinal, renal, and hepatic side effects is well documented, particularly with long-term use (Monteiro-Steagall et al. 2013). In contrast, no adverse events were reported in the PEMF group. This reinforces the utility of PEMF as a non-invasive, low-risk modality, especially in patients with comorbidities or contraindications to NSAID therapy. The only consideration that may arise in the use of this therapy is its actual feasibility in an owner’s daily routine: the need to repeat sessions for several weeks could pose a logistical challenge for some caregivers, particularly in an outpatient setting, although during the course of this study there appeared to be a high level of acceptance and tolerance of PEMF in both animals and caregivers.

Taken together, the results of this study contribute to the growing body of evidence supporting PEMF as a viable and effective therapy for canine OA. The benefits observed in pain control, muscle preservation, and joint mobility justify its consideration as part of a multimodal treatment strategy, thereby potentially reducing reliance on long-term NSAID administration.

A potential limitation of our study is the relatively small sample size, which may have reduced the power to detect statistically significant differences in some parameters, such as gait analysis outcomes. The short follow-up period limits the ability to assess long-term outcomes and OA progression. In addition, no blinding of owners or outcome assessors was implemented, which may have introduced assessment bias, particularly for subjective measures such as pain and lameness scores. Future multicentre studies with larger cohorts, extended follow-up periods, and blinded assessment are needed to validate and expand upon these preliminary findings.

This pilot study suggests that pulsed electromagnetic field (PEMF) therapy may provide clinical benefits comparable to those of a standard NSAID protocol in the management of canine osteoarthritis. In our cohort, PEMF appeared to offer earlier and more sustained reductions in pain and lameness, together with improvements in muscle circumference and joint mobility, without observed adverse reactions or radiographic evidence of disease progression.

However, these findings should be interpreted with caution, given the small sample size and short follow-up period. PEMF may represent a safe and promising alternative or adjunctive option, particularly in dogs with contraindications to pharmacological therapies. Larger, multicentre trials with longer follow-up are warranted to confirm these preliminary results and to establish standardised treatment protocols for routine clinical use.
